# Implementing what works: a case study of integrated primary health care revitalisation in Timor-Leste

**DOI:** 10.1186/1447-056X-13-5

**Published:** 2014-02-24

**Authors:** Nelson Martins, Lyndal J Trevena

**Affiliations:** 1 Faculty of Medicine and Health Sciences, University Nacionale Timor-Leste, Dili, Timor-Leste; 2 Sydney School of Public Health, University of Sydney, Room 321b, Edward Ford Building (A27), Sydney, NSW 2006, Australia

## Abstract

**Background:**

Revitalising primary health care (PHC) and the need to reach MDG targets requires developing countries to adapt current evidence about effective health systems to their local context. Timor-Leste in one of the world’s newest developing nations, with high maternal and child mortality rates, malaria, TB and malnutrition. Mountainous terrain and lack of transport pose serious challenges for accessing health services and implementing preventive health strategies.

**Methods:**

We conducted a non-systematic review of the literature and identified six components of an effective PHC system. These were mapped onto three countries’ PHC systems and present a case study from Timor-Leste’s Servisu Integrado du Saude Comunidade (SISCa) focussing on MDGs. Some of the challenges of implementing these into practice are shown through locally collected health system data.

**Results:**

An effective PHC system comprises 1) Strong leadership and government in human rights for health; 2) Prioritisation of cost-effective interventions; 3) Establishing an interactive and integrated culture of community engagement; 4) Providing an integrated continuum of care at the community level; 5) Supporting skilled and equipped health workers at all levels of the health system; 6) Creating a systems cycle of feedback using data to inform health care. The implementation case study from Timor-Leste (population 1 million) shows that in its third year, limited country-wide data had been collected and the SISCa program provided over half a million health interactions at the village level. However, only half of SISCa clinics were functional across the country. Attendances included not only pregnant women and children, but also adults and older community members. Development partners have played a key role in supporting this implementation process.

**Conclusion:**

The SISCa program is a PHC model implementing current best practice to reach remote communities in a new developing country. Despite limited resources, village level healthcare and engagement can be achieved but takes a long-term commitment and partnership.

## Background

The thirty-year anniversary of the 1978 Alma-Ata declaration called for the revitalisation of Primary Health Care as “a set of guiding values for health development, a set of principles for the organisation of health services, and a range of approaches for addressing priority health needs and the fundamental determinants of health” [1]. The World Health Report in that same year was careful to make the distinction between the service-delivery-focused ‘primary care’ and ‘primary health care’ which more broadly mobilises societies to transform health systems driven by values such as equity, solidarity and participation [2].

Whilst the Alma-Ata Declaration did not see the achievement of health for all by 2000, the rebirth of many of these ideals within the broader cross-sectoral development agenda in the Millennium Development Goals (MDGs) has continued a level of commitment, arguably at a greater level. However, as the world sits a few years away from the MDG target date of 2015, there has been patchy achievement, particularly in the health-related goals MDG 4,5 and 6 worldwide [3] and the focus in many countries continues to be on vertically-driven, selective programs and donor-driven agendas.

Current thinking advocates for integration, a continuum of care approach and closer linkage of health to development [1]. This has been accompanied by a growing body of knowledge about what has worked and what has not. Encouragingly, the evidence supports a number of the principles and components which were part of the original Alma-Ata Declaration but the challenges of implementation remain. In this paper we summarise the evidence-base for six core principles and components of effective primary health care and provide a case example from Timor-Leste of how they have been implemented.

## Method

We conducted a non-systematic review of the literature using key words primary healthcare, health outcome and systematic review in Medline, the Cochrane Database of Systematic Reviews, DARE and Health Technology Assessment. To try and capture reports in the grey literature we also search Google and key international development agency websites. We used a series of articles published in the Lancet for the 30-year anniversary of the Alma Ata Declaration as a core set of papers and hand-searched the bibliographies of these and related articles. Articles were included if they provided some level of systematic review of evidence relating to PHC in low or middle-income countries. This case study does not include any experimental research, nor any research carried out on humans or animals and as such did not require ethics committee approval.

**Figure 1:**
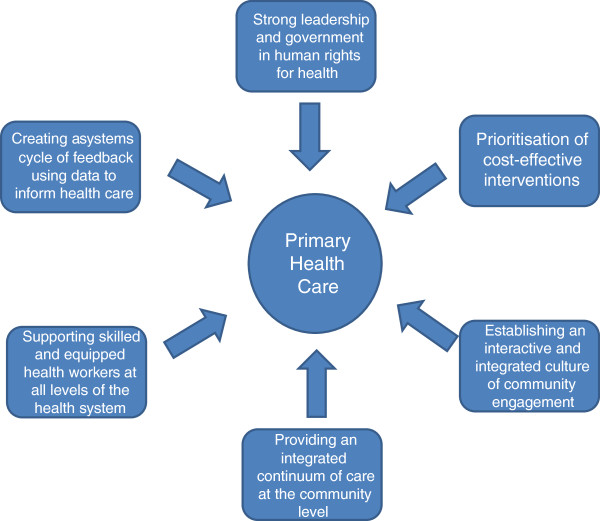
Core principles & components for effective implementation of primary health care.

A case study of the implementation of PHC revitalisation was documented by the first author of this paper (NM), the former health minister and developer of the Servisu Integrado du Saude Comunidade (SISCa) program. The second author was an independent observer of the program over a four month period. Since the SISCa concept had been adapted partly from the Cuban and Indonesian systems we also mapped the PHC revitalisation strategies onto these three country systems. Finally, we were able to obtain locally collected data about the extent of SISCa implementation after three the first three years (Figure 1).

## Results

### Six components of an effective primary health care system

#### 1. Strong leadership, partnership and government in human rights for health

The World Health Organisation’s constitution states that “the enjoyment of the highest attainable standard of health is one of the fundamental rights of every human being”. The protection of human rights for health can only be achieved through inter-sectoral action requiring a ‘whole of government’ commitment [4]. Social development and good governance are linked to strong comprehensive primary health care systems [5]. These require leadership and commitment to appropriate strategies and funding by national governments. A review showed that of the fourteen countries furthest along the path of progress towards a comprehensive and equitable primary health care system, almost all have benefited from strong government commitment to an agreed national essential health package with defined priorities and links to the not-for-profit sector, non-government organisations and other service providers in the system [5].

#### 2. Prioritisation of cost-effective interventions

There is a growing body of evidence for cost-effective interventions which can be implemented at the primary care level in developing countries. A pragmatic coverage of these interventions in the maternal, neonatal and child health area, would prevent 20-30% of maternal deaths, 20-21% neonatal deaths and 29-40% of post-neonatal deaths [6]. If 99% coverage of this package is achieved, their model predicts that this would increase to 67.3% of maternal deaths, 45% neonatal and 79% of post-neonatal deaths averted.

The list of twenty seven proven interventions in Table 1 provides a road-map for countries who, under the MDG targets aim to reduce child mortality by two-thirds, reduce the maternal mortality ratio by three-quarters and to have halted and begun to reverse the incidence of HIV/AIDS, malaria and others diseases (including TB). Prioritising these interventions clearly within a nation’s health strategy and planning is clearly important to achieving these outcomes.

**Table 1:** MDG-related cost-effective interventions for primary health care in developing countries

***Cost-effective interventions:***	**MDG Goals**
-Promotion of reproductive health and family planning	4,5
-Promotion of appropriate care-seeking and antenatal care in pregnancy	4,5
-Promotion of skilled care for childbirth	4,5
-Exclusive breastfeeding advice and support	4
*Preventive interventions:*	
-Provision/availability of contraceptives for birth spacing	1,4,5
-Cord care and clean delivery kits	4,5
-Iron, folate or multiple micronutrient supplementation in pregnancy	4,5
-Balanced protein-energy supplements during pregnancy in food-insecure populations	1,4,5
-Calcium supplementation for PIH	4,5
-Low dose aspirin in high risk pregnancies	4,5
-Anti-retrovirals in HIV-infected individuals and PMTCT	4,5,6
-Antibiotics for premature rupture of membranes	4,5
-Antenatal steroids for those at risk of pre-term birth	4,5
-EPI (including new vaccines for HIB, pneumococcal and rotavirus)	4
-Vitamin A supplementation in children	4
-Zinc supplementation in children for prevention of diarrhoea and pneumonia	4
-Insecticide treated bed-nets for family	4,5,6
-Intermittent preventive treatment for malaria in pregnant women and children (IPT)	4,5,6
-*Household-level water storage and disinfection*	4,5,6,7
*Treatment interventions:*	
-Promotion and use of skilled birth attendants at health facilities	4,5
-Interventions for prevention of post-partum haemorrhage and use of oxytocics.	4,5
-Basic newborn resuscitation with bag and mask	4
-Improved diarrhoea management (zinc and ORT)	4
-Community detection and treatment of pneumonia with short course amoxicillin	4
-Improved case management of malaria (including ACTs)	6
-Recognition, triage and treatment of severe malnutrition in affected children in the community setting	1,4
-*Active case identification of TB in households and treatment with DOTs	6

Adapted from Bhutta et al. [6].

*denotes additional new items of relevance to Timor-Leste context.

#### 3. Establishing an interactive and integrated culture of community engagement

Community engagement goes to the heart of the principles of Alma Ata and yet, has often been the component of primary health care that has been most neglected. Within this construct of ‘community engagement’ it’s important to distinguish between ‘participation', ‘mobilisation’ and ‘empowerment’. Participation can either be active or passive involvement whereas ‘mobilisation’ usually refers to communities responding to directives from health professionals to improve their health. More recently, there has been an important return to ‘empowerment’ strategies that have health workers acting as facilitators with communities to identify strategies and make decisions that impact on the ‘process’ of health improvement [7,8].

Community-mediated interventions can improve health outcomes although many of these have been top-down strategies through training community health workers and less often through bottom-up or interactive models of community engagement. A Cochrane review of eighteen trials showed that community-based interventions did not show any reduction in maternal mortality (RR 0.77; 95% CI 0.59 to 1.02) but did significantly reduce maternal morbidity (RR 0.75; 95% CI 0.61 to 0.92), neonatal mortality (RR 0.76; 95% CI 0.68 to 0.84), stillbirths (RR 0.84; 95% CI 0.74 to 0.97), and perinatal mortality (RR 0.80; 95% CI 0.71 to 0.91). It also increased the referrals to health facilities for pregnancy-related complications by 40% (RR 1.40; 95% CI 1.19 to 1.65), and improved the rates of early breastfeeding by 94% (RR 1.94; 95% CI 1.56 to 2.42) [9]. A separate review by Bhutta et al. found the pooled effect of community-based interventions resulted in a 31% reduction in neonatal mortality (RR = 0.69, 95%CI 0.61-0.77), a 29% reduction in peri-natal mortality (RR = 0.71, 95% CI 0.61-0.84) and 29% reduction in maternal morbidity (RR = 0.71, 95% CI 0.53-0.94) [6].

Unfortunately, early evidence for the effectiveness of community empowerment strategies was largely replaced by an exclusive focus on evidence-based cost-effective interventions whose effects could be more easily quantified in the 1990s and early 2000’s [8]. The research and global health community needs to embrace new research methodologies which measure the processes of decision-making and empowerment in communities. Such strategies have the potential to not only improve health literacy, but also to address local cultural issues and health beliefs as well as geographic and health challenges specific to that community [10]. Studies which develop and evaluate complex interventions to facilitate informed decision-making for communities in developing country settings may help to improve understanding of this in the future [11].

#### 4. Providing an integrated continuum of care at the community level

Several important analyses have recently looked at what has worked and what has not. Rohde et al. took life expectancy against gross national income (GNI) and identified that the top thirty low and middle income countries could be categorised as having one of three types of primary health care systems - selective primary health care, primary health care in transition, or comprehensive primary health care [5]. Selective primary health care systems focussed on specific interventions delivered vertically but at the community level. Comprehensive primary health care systems on the other hand, have a strong focus on community health workers, good referral systems to the district level, equitable access to services for all, including the poorest communities and those geographically isolated and the use of data for decision-making. These countries were more likely to have more skilled birth attendances, lower maternal and child mortality, higher contraceptive prevalence, better immunisation coverage and inter-sectoral outcomes such as access to safe drinking water, school attendance by girls and adult literacy levels [5]. They are also associated with improved effectiveness, equity and efficiency [12].

Comprehensive primary health care systems should be best placed to implement a ‘continuum of care’ across the lifecycle. Maternal and child health advocates have recently described how this might look in practice by aiming for universal coverage with integrated essential care packages across the maternal and child health lifecycle and alongside other strategies such as malaria, TB, water and sanitation programs [13]. Whilst the association between better health and comprehensive primary health care is encouraging, a separate review comparing the effect of integrated services against fragmented services was inconclusive and unable to find enough experimental evidence to infer causality [14].

#### 5. Supporting skilled and equipped health workers at all levels of the health system

There are many challenges in retaining and supporting health workers which can threaten emerging primary health care systems. Efficient systems and good governance must ensure appropriate financing and distribution of essential medications and equipment in a sustainable system. Effective and clear referral pathways must be available for serious clinical cases.

However, developed world experience shows that simply having staff and systems in place does not necessarily translate evidence into practice. Cost-effective interventions exist and a number of international protocols have been translated and adapted for country-specific contexts. However, the real challenge lies with the effective implementation of such interventions, guidelines and protocols. A review of four randomised and six non-randomised controlled studies in developing countries looking at the effect of education and training on health professional behaviour. These showed an improvement in compliance with guidelines but the effect was not sustained. A range of educational formats, including locally adapted and facilitated sessions were used but none were compared head-to-head. There is insufficient evidence to assess the effectiveness of educational outreach, local opinion leaders, use of mass media, and reminders. Educational materials alone are unlikely to influence change [15]. Creating effective ways of keeping health professionals up-to-date in developing countries is an important item for the future research agenda.

#### 6. Creating a systems cycle of feedback using data to inform health care

Feedback with data to communities and to health professionals is likely to improve the implementation of effective strategies into practice [15]. In fact, this is the only strategy that has been shown to have such an effect in developing countries through one randomised and three non-randomised controlled studies (all hospital-based). Several of the studies highlighted that sustainability can be limited by organisational barriers. One suggested framework for the implementation of an integrated primary health care strategy puts data collection and feedback as a vital part of an effective health system. This requires governments to support their district managers to implement, monitor and evaluate their health services and to disseminate the results. At the central and district health sector level, the use of data in the process of planning and monitoring policy and service delivery is challenging but has the potential to focus limited resources on identified gaps and to highlight areas of success. At the service provision and community level it can also be used for audit and feedback for quality improvement for health providers and for empowerment of communities [16].

### Implementing the core components of PHC revitalisation in practice: A case study from Timor-Leste

Timor-Leste (formerly known as East Timor) lies on the eastern side of the island of Timor, with the western half being part of Indonesia. It is located to the north of Australia and west of Papua. The total population is approximately one million, with the majority of people living in remote villages (sucos and aldeias) in rugged mountain terrain, often cut-off by landslides and flooded rivers during the wet season. From the 16th century onwards, Timor-Leste was a Portuguese colony, occupied briefly by Japan in World War II and defended by Australian and Dutch forces. There was a brief period of semi-independence in mid-November 1975 but Timor-Leste was invaded by Indonesia on 7 December 1975 and occupied until the UN took over in 1999 and subsequently became a formally independent nation on May 20, 2002 after UN-supervised elections in 2001. The stability of Timor-Leste in the first few years of democracy was fragile with a serious conflict breaking out in 2006 and many people being internally displaced. Only since 2007 has there been a period of stability and economic progress. Timor-Leste has oil and gas reserves managed in trust by the World Bank which provide a regulated income source for the nation.

### Structure of the overall health system and health-seeking behaviour

**Figure 2:**
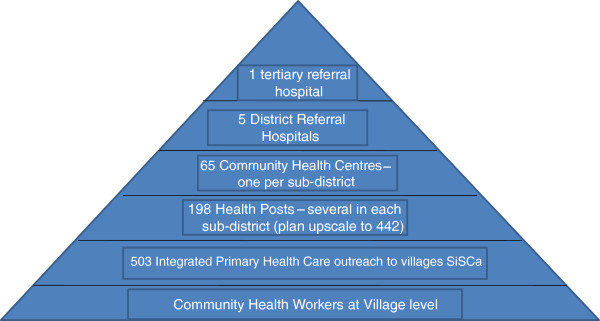
Overall structure of the Timor-Leste Health System.

During the past ten years the Government of Timor-Leste has established a tiered health system with the following basic structure (Figure 2).

Most effort has been on the establishment of core infrastructure, human resource planning and placement across the health system. There are currently 2.2 nurses per 1,000 population and 0.1 doctors per 1,000 population [17]. The medical workforce was instantly supplied in 2004 by the Cuban government through a bilateral agreement supplying several hundred Cuban doctors to Timor-Leste and the training of one thousand Timorese doctors, initially in Cuba and now at the National University of Timor-Leste (UNTL). These Timorese graduates are now starting to work within the Timorese health system. International technical advisors in the Ministry of Health are gradually being phased out and local policy development is exemplified by the recent development of the new National Health Strategy of Timorese Policymakers.

The 2003 Timor-Leste Demographic Health Survey reported that one-quarter of the population reported that it took more than two hours to reach their usual health provider and 86% walked to their nearest health facility. By 2008 a national study of health-seeking behaviour reiterated that participants still described long and difficult journeys to seek assistance for illness, using locally available traditional remedies as an alternative with limited capacity to engage in preventive health behaviours [18]. Although the Ministry of Health established one community health centre (CHC) with moderate in-patient facilities in each of the thirteen districts, and one CHC with no in-patient capacity in each sub-district, there was not the capacity to reach one health post in every village cluster (suco) with only 198 of the planned 442 established. One of the key reasons for developing the SISCa program was to address this shortfall at the community level, improving uptake of preventive healthcare and access to basic medical services.

### Development of the SISCa (Servico Integrado da Saude Comunitaria) and coverage

In 2008, the SISCa program commenced with each sub-district CHC required to deliver the SISCa program monthly in every suco (village), usually in an outdoor meeting area, or a local resident’s home. The team includes a doctor, midwife, health promotion officer, nurse and/or lab technician depending on available health workforce. During this early implementation period, support is also provided by non-government organisations who act as development partners in strengthening the health system. The model has similarities to the Indonesian Posyandu program [19] but provides a more comprehensive primary health care model as show in Table 2. It has also borrowed elements from the Cuban system which includes active case finding, home visitation and community health registers. Table 2 shows how these three country examples map across the six components of effective primary health care outlined in the first part of this paper.

**Table 2:** Implementation of effective PHC components in remote communities - SISCa, Posyandu and Cuban systems

**Principles and components of effective primary health care**	**SISCa program in Timor-Leste**	**Posyandu program in Indonesia**	**Cuban primary health care system**
**Values**			
**Component 1**			
Strong leadership and government in human rights for health	Health as a human right in Timor-Leste’s constitution since 2002. Free basic healthcare for all citizens	Government support for health as a basic human need to live a productive life. Primary care free via social insurance scheme (if eligible)	Post-revolutionary socialist government responsible for healthcare as a human right and free for all citizens
**Component 2**			
Establishing an interactive and integrated culture of community engagement	Community empowerment through community health workers (PSF), women’s self-help groups and village councils	Use of community volunteers (cadres) to provide support to communities	Active community participation encouraged in health system through family doctor outreach as a joint social responsibility
**Structural**			
**Component 3**			
Prioritisation of cost effective interventions	Six tables targeting MDGs 1,4,5,6,7 but also providing some comprehensive ambulatory care via monthly outreach clinics in villages (sucos)	Five tables targeting maternal and child health (MDGs 1,4 & 5) via monthly clinics at community healthcare post	Comprehensive primary healthcare (family & preventive medicine, inter-sectoral action) mainly via family doctors based at community clinics but who also live in the communities.
**Component 4**			
Provide an integrated continuum of care	Comprehensive coverage of maternal & child health, active case finding and home visits including TB, leprosy, malaria control to whole community. General ambulatory care for all ages including chronic disease management. Occasional outreach specialist care (eg dental, eyes)	Outreach clinic focus on maternal and child health	Doctor-led health team in local polyclinic. Active case-finding and home visitation from these facilities. High coverage of health facilities in remote areas.
**Functional**			
**Component 5**			
Supporting skilled and equipped health workers at all levels of system	Healthcare delivery and referral at outreach clinics by doctors, midwives, nurses and health promotion staff with support of NGOs	Village midwife and immunisation nurse deliver MCH program with supervision of doctor from sub-district clinic	High ratio of doctors per community, with responsibility for local health outcomes
**Component 6**			
Create a systems cycle of feedback using data to inform healthcare	A ‘library’ of register books for each community	Data collection and feedback not systematic	Local register books of community health data systematically collected and maintained by family doctors

There are six components (tables) within the SISCa structure.

### Table one: Registration

*Family registration* (Registo Saude Familiar): Each suco should have a register of all households to be used to monitor key health indicators nationally and locally. The data include names and ages of all household members, number of pregnant women, chronic diseases, children with malnutrition, immunisations, TB cases, recent births and any deaths in the household. It forms the denominator for the ‘library of registers’ which are an important functional component of the SISCa program (described later in this article). Once the family register is completed it can be updated every month by the local health team who should be aware of new pregnancies, births, illnesses and deaths.

### Table two: Nutrition assistance and child health promotion

Malnutrition is a significant problem in Timor-Leste with almost half of children under five years stunted, underweight or malnourished. Mothers are encouraged to bring all children under-five to be weighed and have their mid-upper-arm circumference measured. Counselling and referral for under-nutrition is integrated with this service. Exclusive breastfeeding and good maternal nutrition is encouraged. Children are offered Vitamin A and de-worming treatment every six months and childhood immunisations are administered. Potential agricultural solutions to food insecurity should be explored and mothers taught how to cook healthy meals for young children using local foods through meetings with the local leaders, cooking demonstrations and posters or flyers with pictures of healthy food options. Food supplements and supplies can be distributed to families at risk. Inter-sectoral collaboration with agriculture can be facilitated through the SISCa program’s community engagement and surveillance.

Timor-Leste has seen infant mortality fall from 68 per 1,000 live births in the period 1999–2003 [20] to 44 per 1,000 live births in 2004–2009 [21]. The MDG4 target for under-five mortality by 2015 is 53 per 1,000 live births and the 2010 Demographic Health Survey (DHS) reported it is currently 64 per 1,000 live births. There has also been an almost threefold increase in the number of children 12–23 months considered by the WHO to be “fully immunized”, from 18 percent in 2003 to 53 percent in 2009 [21] and a recent report by the UNDP goes so far as to suggest that these improvements could be attributable, at least in part, to the SISCa program [22]. Ministry of Health routinely collected data in 2010 suggests this immunisation coverage may have risen further to around 66 per cent [23]. Routinely collected data also shows that the proportion of children under five being weighed has increased from 14.1 per cent in 2008 to 21.2 per cent in 2010 [23]. The SISCa program is providing a platform for a more assertive effort to address this problem.

### Table three: Maternal health and family spacing

The MDG5 target for reduced maternal mortality may not be achieved by 2015 with a maternal mortality ratio of 557 per 100,000 [24] and the Ministry of Health reporting 450 per 100,000 live births [23]. Midwives are in attendance if possible, and a register is maintained of all pregnancies. The first half of the patient held record book (LISIO) is completed for pregnant mothers and the second half is for the infant and child. A proactive approach is taken to identifying pregnant women within the community and encouraging them to attend the SISCa for antenatal care. The health team also actively seeks out women who may have delivered recently and require a postnatal check which may involve home visits. A range of family spacing options are available alongside health promotion videos and other resources which seek to facilitate informed choice in family spacing.

The proportion of women having at least one antenatal care increased from 61 percent in 2003 to 86 percent in 2009 and there has been an increase in skilled birth attendance from 18.4 per cent in 2003 to 29.9 per cent [20,24]. The proportion of women receiving postnatal care has also increased from 14.6 per cent to 32 per cent in the same period, fertility rates have fallen from 7.8 to 5.5 and contraceptive coverage has increased from 10 per cent to 22.3 percent.

### Table four: Hygiene, sanitation and malaria prevention

Health promotion staff, community health workers (PSFs), NGOs and others work with communities on personal hygiene, bed-net promotion, vector control and water and sanitation strategies. Hand-washing demonstrations and bed-net distribution to all households of pregnant women and children under five are important responsibilities of this table. Community health workers are also asked to complete a household survey (called Kubasa) on water, sanitation and hygiene prior to the SISCa. As with the other surveys and registers these form a ‘library’ or virtual database for each community.

Access to clean drinking water and adequate sanitation remains a major problem for Timor-Leste. The number of households using an improved water source only increased from 53 per cent to 63 per cent between 2003 and 2009 and household with inadequate sanitation reduced from 70 per cent to 57.1 per cent [20,24]. The number of children under five sleeping under a bed-net has increased from 21.2 per cent in 2007 to 45.5% in 2009 [22].

### Table five: Ambulatory primary care

A doctor or nurse is available to assess sick children and adults and can prescribe basic treatments or refer to secondary care services as required. Treatment of childhood respiratory disease, gastroenteritis, implementation of IMCI, case detection of malaria and TB including spot sputum collection and rapid-tests for malaria and the commencement of treatment or referral can occur. Active case detection of TB and malaria has seen the national case detection rates increase from 76 per cent in 2005 to 84 per cent and cohort cure rates rise from 61 per cent in 2005 to 73 per cent in 2008 [25]. Ministry of Health data also suggests that malaria incidence has reduced with 132.9 cases per 1,000 in 2008 and 104.2 per 1,000 in 2010 [23].

### Table six: Health promotion activities

Activities may include showing films, using flipcharts, group discussions or one-to-one counselling on issues that are relevant to the community (eg good child nutrition, malaria prevention, management of gastroenteritis in children, birth spacing methods, promoting birth at health facilities etc.).

#### SISCA Functional elements (A ‘library’ of registers for each community, Health care and delivery Audit and feedback for quality improvement)

The collection of registers for each community provides a rich dataset to inform health planning and services. Timor-Leste has poor or non-existent internet coverage in most districts, necessitating manual book registers for the moment. It is hoped that eventually this ‘library of registers’ will become electronic linking peripheral centres with each other the central facilities.

SISCa participation rates and evaluations can be fed back to health workers and communities affording an opportunity to change and improve service quality and respond to community needs. For example, local teams may identify poor child nutrition rates as a significant local issue allowing discussion about local solutions and ongoing monitoring of the impact. These functional elements have been amongst the most challenging to implement in a low tech environment with limited staff and community workers with lower levels if numeracy and literacy. It will take time to instil a culture of audit and feedback and to empower communities and local health teams in this way.

### SISCa coverage and impact on health-seeking behaviour

**Figure 3:**
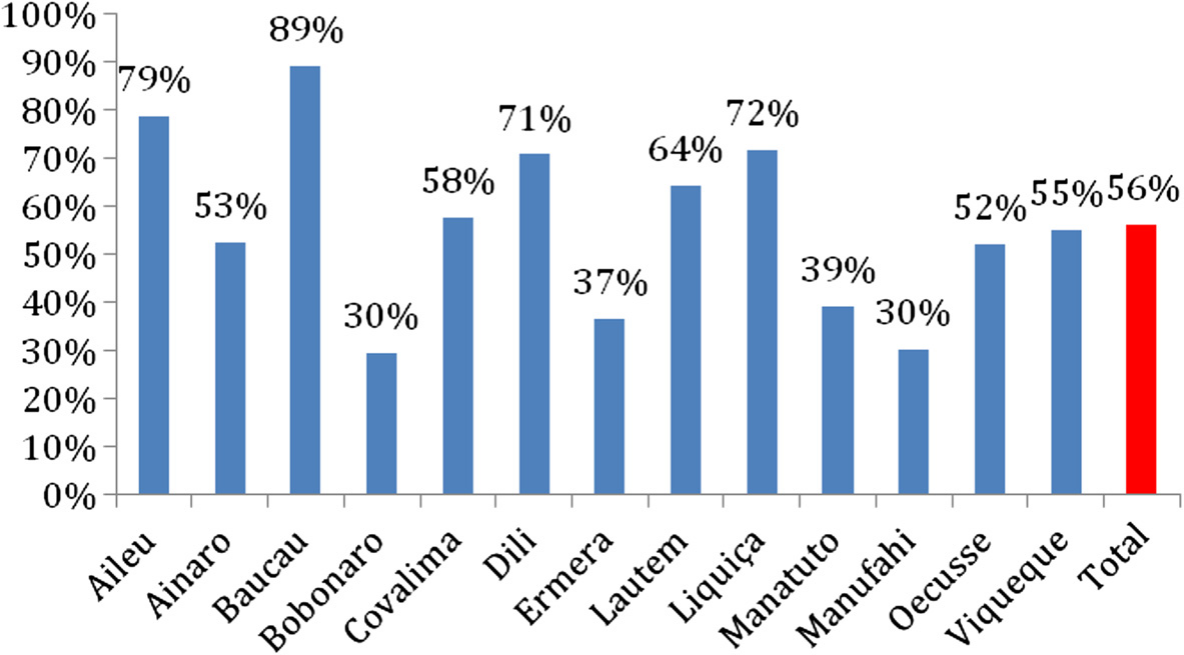
Proportion of monthly SISCa clinics functioning by district 2010.

**Figure 4:**
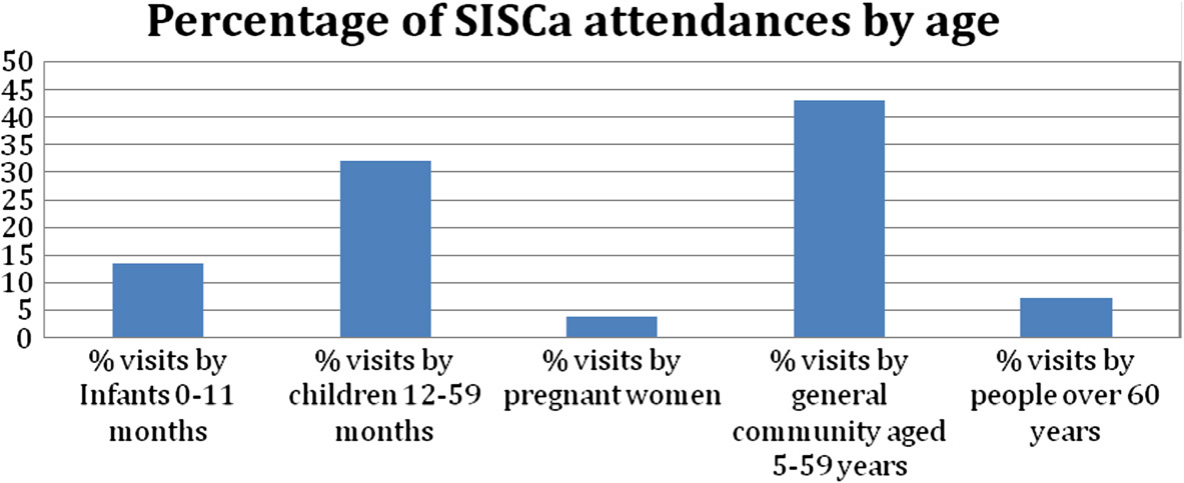
Distribution of SISCa attendees by age 2010 (Total attendances 555,608).

**Figure 5:**
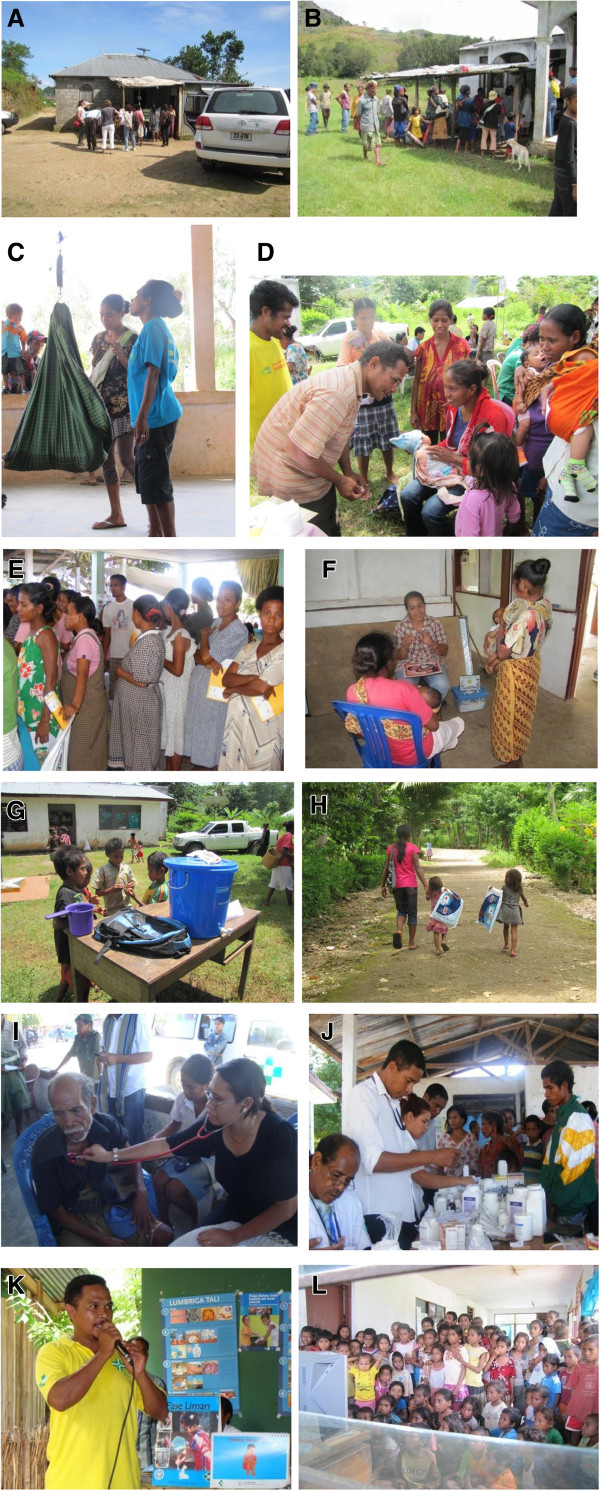
**SISCa activities operating in the community.** Photos **A & B**: Typical SISCa locations. Photos **C & D**: Community worker weighs an infant and a nurse immunises a young baby. Photos **E & F** Pregant women wait for ANC and a family spacing consultation with nurse. Photos **G & H**: Hand-washing and bed-net distribution. Photos **I & J**: Basic medical services and a simple dispensary. Photos **K & L**: Health promotion activities using flipcharts, posters and films.

The SISCa program was specifically designed to revitalise primary healthcare in an equitable fashion following ongoing surveys showing difficulties accessing health facility-based services and poor implementation of preventive healthcare strategies. Challenges remain with health workforce shortages persisting in some areas and many communities being isolated by flooded rivers in the wet season. In 2010, Ministry of Health data shows SISCa coverage varied between 30 and 89% per cent across the country (see Figure 3) but over half a million visits were made to SISCa clinics across the year. The SISCa attendances reflect access to a comprehensive range of healthcare services with a substantial number of older children and adults attending in addition to mothers and babies. (See Figure 4) Although pregnant women were a small proportion of those seen at SISCa clinics in 2010, the 2009–2010 Demographic Health Survey reports that 55.10% pregnant women received at least four antenatal visits. Further evaluation needs to explore whether women prefer and choose to go to health facilities for their antenatal care than at the SISCa clinic and to what extent the SISCa program has allowed a relationship to develop between midwives and women within these remote communities. As the coverage of SISCa clinics improves it will be important to build on this framework of improved access to healthcare through better quality care and using the data to inform at all levels of the system (Figure 5).

### Implementing the six components of effective primary health care

During the first few years of implementation of the SISCa program, some of the components of effective primary health care have been easier to achieve than others. Strong leadership and the inclusion of health as a human right in the country’s constitution have been fundamental in getting the program started and sustained. The SISCa program has remained as a core component in the new 20 year National Strategic Development Plan. The framework of SISCa allows for the prioritisation of many of the cost-effective interventions listed in Table 1, although not everything on this list is part of national protocols and, like most countries, there are substantial variations in the implementation of these. Engagement with communities has been reflected in the attendance rates shown in Figure 4 but the role and sustainability of community volunteers continues to need strengthening. Integrated healthcare is at the core of the SISCa program and has been more widely achieved than other components through the structural components of SISCa. The health workforce in Timor-Leste continues to expand and up-skill with strengthened referral pathways and supply chains being identified as a priority in the new strategic plan. Data systems and feedback cycles will likely be the most challenging component of the system to implement (Figure 3 and 4).

## Conclusion

This paper has identified six core components of an effective PHC revitalisation. It has described how Timor-Leste has extended their primary health care system to integrate basic health service package delivery with active health promotion in community outreach. Although it is not possible to infer causality, the current improvements in health service utilisation, immunisation coverage and reduction in TB and malaria are encouraging and serve to illustrate how the SISCa concept aims to reach remote mountainous communities with targeted and effective interventions. The recently published UN Human Development Report for Timor shows the country is on track for many of the health MDGs with the need for a more concerted effort in measles vaccine coverage, maternal mortality, HIV/AIDS education and malaria control. Strengthening the current system will hopefully help to achieve these targets which will be a major achievement for one of the world’s youngest independent nations.

## Competing interests

Both authors declare: no support from any organisation for the submitted work; no financial relationships with any organisations that might have an interest in the submitted work in the previous three years, no other relationships or activities that could appear to have influenced the submitted work.

Dr Nelson Martins is the former Health Minister for Timor-Leste but has no commercial or other conflicts of interest with the development of the SISCa program. A/Professor Trevena is a primary care and public health academic who has collaborated with the Ministry of Health, Timor-Leste on health systems strengthening and capacity building activities over the past four years. She has no commercial or other conflicts of interest with the SISCa program or with any other aspect of this manuscript.

## Authors’ contributions

NM is the key developer of the Serviso Integrado de Saude Comunitaria (SISCa) concept and has led its implementation over the past four years. He has met with A/Prof LT regularly and discussed and jointly decided on the scope and outline of the paper. He provided detailed content for the SISCa component of the paper and has contributed to multiple revisions of the manuscript. A/Prof LT has completed and synthesised the major evidence review on effective primary health care for this paper and has worked jointly with NM on writing and revising the manuscript from its inception. She has coordinated and sourced much of the data which supports the case study. She will be the corresponding author on the paper. Both authors read and approved the final manuscript.

## References

[B1] Lawn J Rohde J Rifkin S Were M Paul V Chopra M Alma-Ata 30 years on: revolutionary, relevant, and time to revitalise Lancet 2008 13 917 927 10.1016/S0140-6736(08)61402-6 18790315

[B2] WHO World Health Report: Primary Health Care - Now more than ever Book World Health Report: Primary Health Care - Now more than ever 2008 Geneva: World Health Organisation http://www.who.int/whr/2008/en/ (Accessed 11/02/2014)

[B3] UN Millenniun Development Goals Report Book Millenniun Development Goals Report 2010 New York: United Nations http://www.un.org/millenniumgoals/reports/shtml. (Accessed 11/02/2014)

[B4] Braveman P Gruskin S Poverty, equity, human rights and health Bull World Health Organ 2003 13 539 545 12973647 PMC2572503

[B5] Rohde J Cousens M Chopra M Tangcharoensathien V Black R Bhutta Z Lawn J 30 years after Alma-Ata: has primary health care worked in countries? Lancet 2008 13 9642 950 961 10.1016/S0140-6736(08)61405-1 18790318

[B6] Bhutta Z Ali S Cousens S Ali T Haider B Rizvi A Okong P Bhutta A Black R Interventions to address maternal, newborn, and child survival: what difference can integrated primary health care strategies make? Lancet 2008 13 972 989 10.1016/S0140-6736(08)61407-5 18790320

[B7] Freire P Pedagogy of the Oppressed 1970

[B8] Rosata M Laverack G Grabman L Tripathy P Nair N Mwansambo C Azad K Morrison J Bhutta Z Perry H Community participation: lessons for maternal, newborn, and child health Lancet 2008 13 962 971 10.1016/S0140-6736(08)61406-3 18790319

[B9] Lassi ZS Haider BA Bhutta ZA Community-based intervention packages for reducing maternal and neonatal morbidity and mortality and improving neonatal outcomes Cochrane Database Syst Rev 2010 13 11 CD007754 doi:10.1002/14651858. CD007754.pub2 21069697 10.1002/14651858.CD007754.pub2

[B10] Trevena L Barratt A Integrated decision making: definitions for a new discipline Patient Educ Couns 2003 13 265 268 10.1016/S0738-3991(03)00047-8 12900097

[B11] Craig P Dieppe P Macintyre S Mitchie S Nazareth I Petticrew M Developing and evaluating complex interventions: the new Medical Research Council guidance BMJ 2008 13 10.1136/bmj.a1655 PMC2769032 18824488

[B12] Kruk M Porignon D Rockers P Van Lerberghe W The contribution of primary care to health and health systems in low - and middle-income countries: a critical review of major primary care initiatives Soc Sci Med 2010 13 904 911 10.1016/j.socscimed.2009.11.025 20089341

[B13] Kerber KJ de Graft-Johnson JE Bhutta ZA Okong P Starrs A Lawn JE Continuum of care for maternal, newborn, and child health: from slogan to service delivery Lancet 2007 13 1358 1369 10.1016/S0140-6736(07)61578-5 17933651

[B14] Briggs C Garner P Strategies for integrating primary health services in middle- and low-income countries at the point of delivery Cochrane Database Syst Rev 2006 CD003318 doi:003310.001002/14651858.CD14003318.pub14651852 16625576 10.1002/14651858.CD003318.pub2

[B15] Siddiqi K Newell J Robinson M Getting evidence into practice: what works in developing countries? Int J Qual Health Care 2005 13 447 453 10.1093/intqhc/mzi051 15872024

[B16] Ekman B Pathmanathan I Lijestrund J Integrating health interventions for women, newborn babies, and children: a framework for action Lancet 2008 13 990 1000 10.1016/S0140-6736(08)61408-7 18790321

[B17] Langenbrunner J Somanathan A Financing Health Care in East Asia and the Pacific Book Financing Health Care in East Asia and the Pacific 2011 City: The World Bank

[B18] Zwi A Blignault I Glazebrook D Correia V Bateman Steel C Ferreira E Pinto B Timor-Leste Health Care Seeking Behaviour Study Book Timor-Leste Health Care Seeking Behaviour Study 2009 City: The University of New South Wales, Sydney

[B19] Leimena SL Posyandu: a community based vehicle to improve child survival and development Asia Pac J Public Health 1989 13 264 267 10.1177/101053958900300402 2638905

[B20] Ministry of Health & National Statistics Office, University of Newcastle, Australian National University Timor-Leste 2003 Demographic and Health Survey Book Timor-Leste 2003 Demographic and Health Survey 2003 Newcastle, Australia

[B21] Macro I NSD Timor-Leste Demographic and Health Survey 2009–10 Book Timor-Leste Demographic and Health Survey 2009–10 2010 Calverton, Maryland USA: National Statistic Directorate Timor Leste, Ministry of Finance and ICF Macro

[B22] UNDP Timor Leste Human Development Report: Managing natural resources for human development. Developing the non-oil economy to achieve the MDGs Book Timor Leste Human Development Report: Managing Natural Resources for Human Development. Developing the Non-Oil Economy to Achieve the MDGs 2011 New York: United Nations Development Program

[B23] Timor-Leste MH Ministry of Health Data provided by the Ministry of Health, Timor-Leste Book Data Provided by the Ministry of Health 2010 Timor-Leste

[B24] ICF Timor-Leste Demographic and Health Survey 2009–2010: Preliminary Report Book Timor-Leste Demographic and Health Survey 2009–2010: Preliminary Report 2010 Calverton, Maryland, USA: National Statistics Directorate, Ministry of Finance, Democratic Republic of Timor-Leste

[B25] WHO Tuberculosis country profiles: Timor-Leste. World Health organization, geneva 2010 http://www.who.int/tb/country/data/profiles/en/index.html. (Accessed 11/02/2014)

